# The impact of variations in input directions according to ISO 14243 on wearing of knee prostheses

**DOI:** 10.1371/journal.pone.0206496

**Published:** 2018-10-29

**Authors:** Xiao-Hong Wang, Wei Zhang, Da-Yong Song, Hui Li, Xiang Dong, Min Zhang, Feng Zhao, Zhong-Min Jin, Cheng-Kung Cheng

**Affiliations:** 1 School of Biological Science and Medical Engineering, Beihang University, Beijing, China; 2 Beijing Medical Implant Engineering Research Center, Beijing Naton Technology Group Co.LTD, Beijing, China; 3 Beijing Engineering Laboratory of Functional Medical Materials and Devices, Beijing Naton Technology Group Co.LTD, Beijing, China; 4 Beijing Advanced Innovation Center for Biomedical Engineering, Beihang University, Beijing, China; 5 Institute of Tribology, School of Mechanical Engineering, Southwest Jiaotong University, Chengdu, China; 6 Institute of Medical and Biological Engineering, University of Leeds, Leeds, United Kingdom; University of Illinois at Urbana-Champaign, UNITED STATES

## Abstract

ISO 14243 is the governing standard for wear testing of knee prostheses, but there is controversy over the correct direction of anterior-posterior (AP) displacement and loading and the correct direction of tibial rotation (TR) angles and torque. This study aimed to analyze how altering the direction of AP and TR affected wear on the tibial insert. Modifications to the conditions specified in ISO 14243–1 and ISO 14243–3 were also proposed. As such, five loading conditions were applied to FEA models of a knee prosthesis: (1) Modified ISO 14243–3 with positive AP displacement and TR angle, (2) ISO 14243–3:2004 with negative AP displacement and positive TR angle, (3) ISO 14243–3:2014 with positive AP displacement and negative TR angle, (4) Modified ISO 14243–1 with positive AP load and TR torque, and (5) ISO 14243–1:2009 with negative AP load and positive TR torque. This study found that changing the input directions for AP and TR according to ISO 14243–1 and 14243–3 had an influence on the wear rate and wear contours on the tibial insert model. However, the extent of wear varies depending on the design features of the tibial insert and shape of the input curves. For displacement control according to ISO 14243–3, changing the direction of AP displacement had a marked influence on the wear rate (272.77%), but changing the direction of TR angle had a much lower impact (2.17%). For load control according to ISO 14243–1, reversing the AP load (ISO 14243–1:2009) only increased the wear rate by 6.73% in comparison to the modified ISO 14243–1 conditions. The clinical relevance of this study is that the results demonstrate that tibial wear is affected by the direction of application of AP and TR. Incorrect application of the loading conditions during the design stage may lead to an ineffective preclinical evaluation and could subsequently influence implant longevity in clinical use.

## Introduction

The success of total knee arthroplasty (TKA) for returning knee functionality has contributed to its widening application for treating diseases of the knee that have failed conservative treatments [1]. However, even with successive improvements in implant designs and materials, implant failure and patient dissatisfaction still persist [2–5]. Loosening of the implant is the most common reason for requiring a second TKA [6–8], which is reported to be linked to malalignment of the motion axis and the generation of wear particles which can induce osteolysis around the implant [9–10]. Therefore, in vitro wear testing is an important factor in the development of knee protheses and is a key requirement for regulatory clearance of such devices.

**Fig 1:**
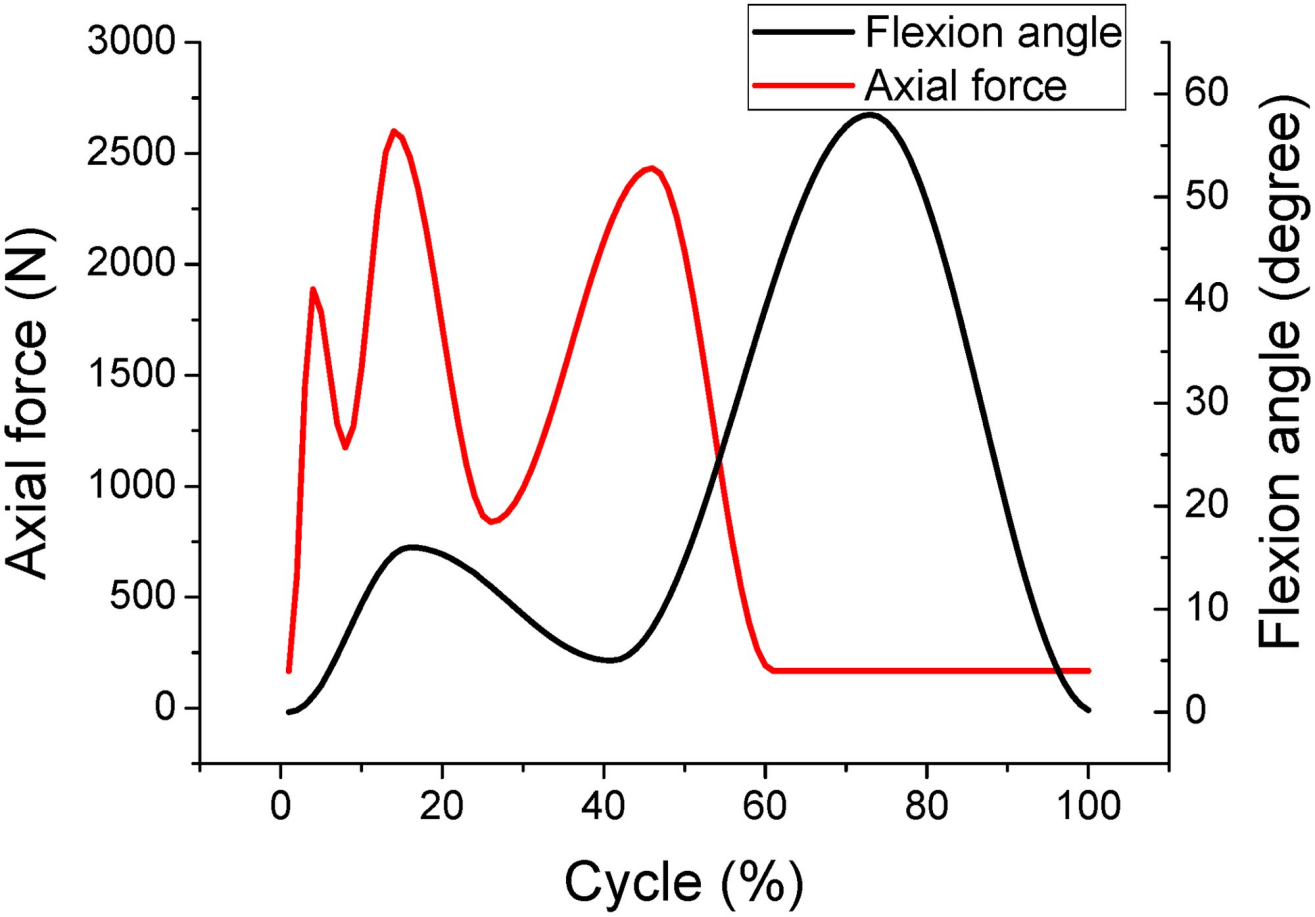
The flexion and axial load inputs are same across the ISO 14243 range of standards.

**Fig 2:**
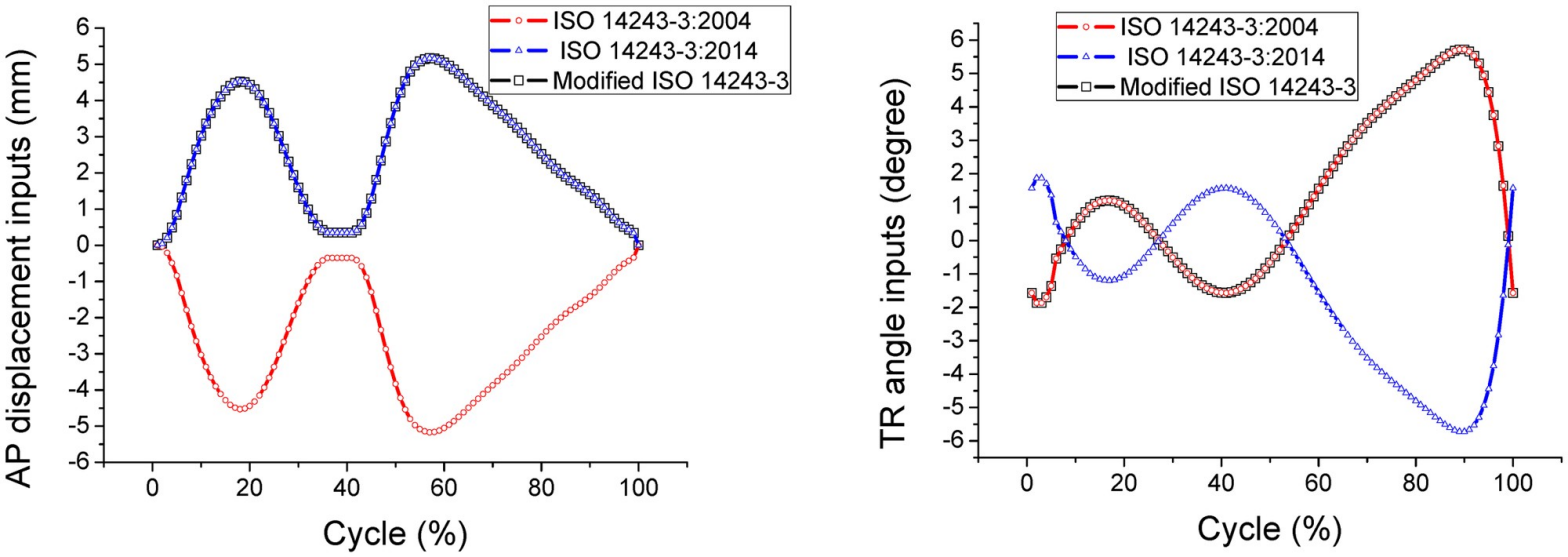
Displacement control: Controversy in the definition of the direction of AP displacement and TR angle in ISO 14243–3. (1) ISO 14243–3:2004: negative AP displacement and positive TR angle. (2) ISO 14243–3:2014: positive AP displacement and negative TR angle. (3) Modified ISO 14243–3: positive AP displacement and TR angle.

**Fig 3:**
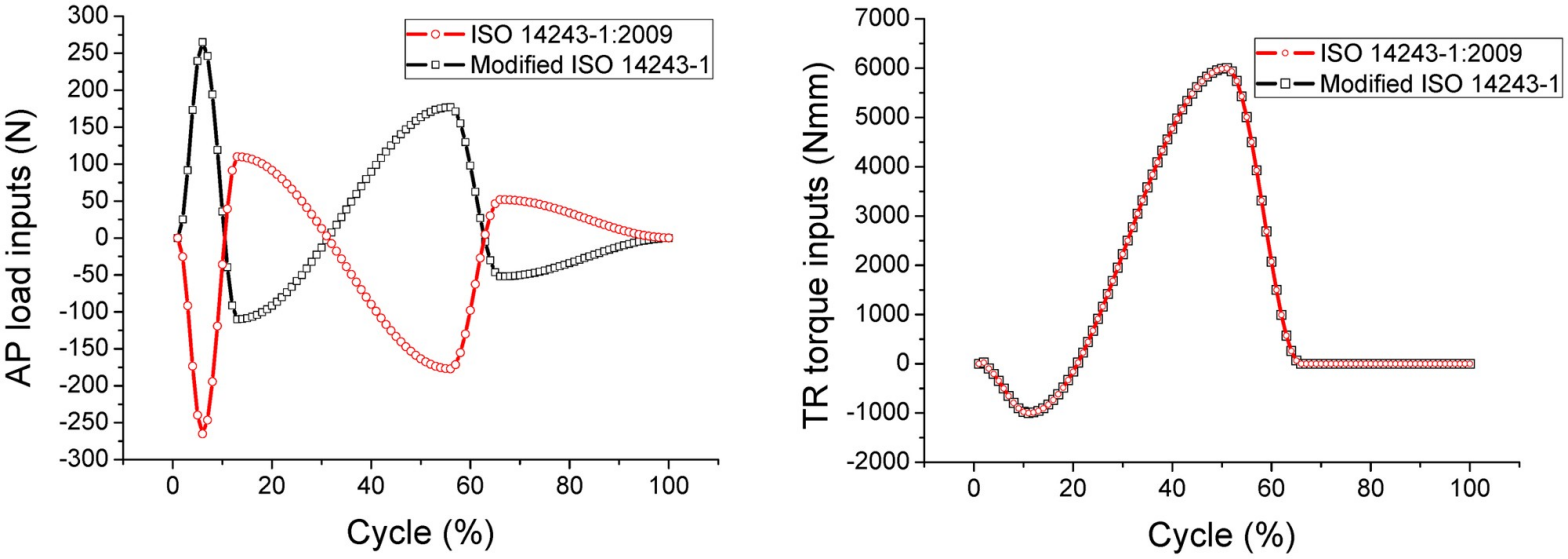
Load control: Controversy in the definition of the direction of AP in ISO 14243–1. (1) ISO 14243–1:2009: negative AP load and positive TR torque. (2) Modified ISO 14243–1: positive AP load and TR torque.

ISO 14243 is the most commonly used standard for evaluating the wear properties of knee implants [11–17]. There are two control modes for the wear test during simulated gait: displacement control according to ISO 14243–3 [11–12], and load control according to ISO 14243–1 [13–14]. The inputs for displacement control are anterior-posterior (AP) displacement and tibial rotation (TR) angle, and the inputs for load control are AP load and TR torque. The knee simulator used to perform the gait movements requires four inputs; flexion angle, axial load, AP displacement or AP load, TR angle or TR torque. The magnitude and direction of the flexion angle and axial load inputs are same across the ISO 14243 range of standards [11–14] (Fig 1). However, there is some controversy around the direction of AP load in ISO 14243–1, and AP displacement and TR angle in ISO 14243–3 (S1 Table). According to ISO 14243 [11–14], the TR angle is positive when the tibia rotates internally and AP displacement is considered to be positive when the tibia moves anteriorly. For illustration, variations in AP and TR are plotted in Figs 2 and 3. A precise and accurate standard for wear performance is crucial for the development of knee prostheses as it predicts the lifetime of the tibial bearing.

ISO 14243–3 is the primary standard for displacement-controlled simulations of wearing of knee prostheses. Compared with the 2004 revision of ISO 14243–3, the 2014 revision reversed the direction of AP displacement and TR angle but maintained the same magnitudes (Fig 2). As seen in Fig 1, the knee flexion angle noticeably increases as the gait cycle moves from 40% to 70%. From Fig 2, in the range of 40% to 70% of the gait cycle, the tibia translates anteriorly and rotates externally according to ISO 14243–3:2014 (positive AP displacement and negative TR angle), but the tibia moves posteriorly and rotates internally according to ISO 14243–3:2004 (negative AP displacement and positive TR angle). However, during normal human gait the tibia undergoes positive AP displacement and rotates through a positive TR angle [18–23]. This current study is proposing a modification to ISO 14243–3:2014 by using positive AP displacement and TR angle (referred to as ‘Modified ISO 14243–3’ in Fig 2).

ISO 14243–1 is the primary standard for load-controlled simulations of wearing of knee prostheses. Typically, when walking, the knee flexes between 5° to 60° and the tibia moves in an anterior direction. This is due to knee flexion and femoral rollback occurring simultaneously [18–23]. From Fig 3, in the range of 40% to 70% of the gait cycle, as the knee is flexed the AP load is acting mainly in the posterior direction according to ISO 14243–1:2009, which is opposite to what actually happens in a normal knee. As above, this study is also proposing a modification to ISO 14243–1:2009 by using positive AP load and TR torque (referred to as ‘Modified ISO 14243–1’ in Fig 3).

A number of studies [24–29] have investigated how varying the parameters outlined in ISO 14243 may impact the knee joint; differences in wear between load and displacement control [24–25], the effect of varying the amplitude of inputs for displacement control on wear [26], the effect of anterior-posterior and internal-external motion constraints on wear [27], the impact of different activities on the wear performance [28], comparison between electromechanically- and pneumatically-controlled knee simulators [29]. To the best of our knowledge, no previous studies considered the influence of altering the direction of AP and TR on wear rates. A misjudgment or incorrect evaluation in this area may lead to an ineffective preclinical evaluation and subsequently influence the longevity of the tibial insert in clinical use.

This study aimed to analyze the influence of changing the directions of AP load in ISO 14243–1, and AP displacement and TR angle in ISO 14243–3 by using kinematic analysis and wear evaluation of knee implants.

## Materials and methods

### Materials

**Fig 4:**
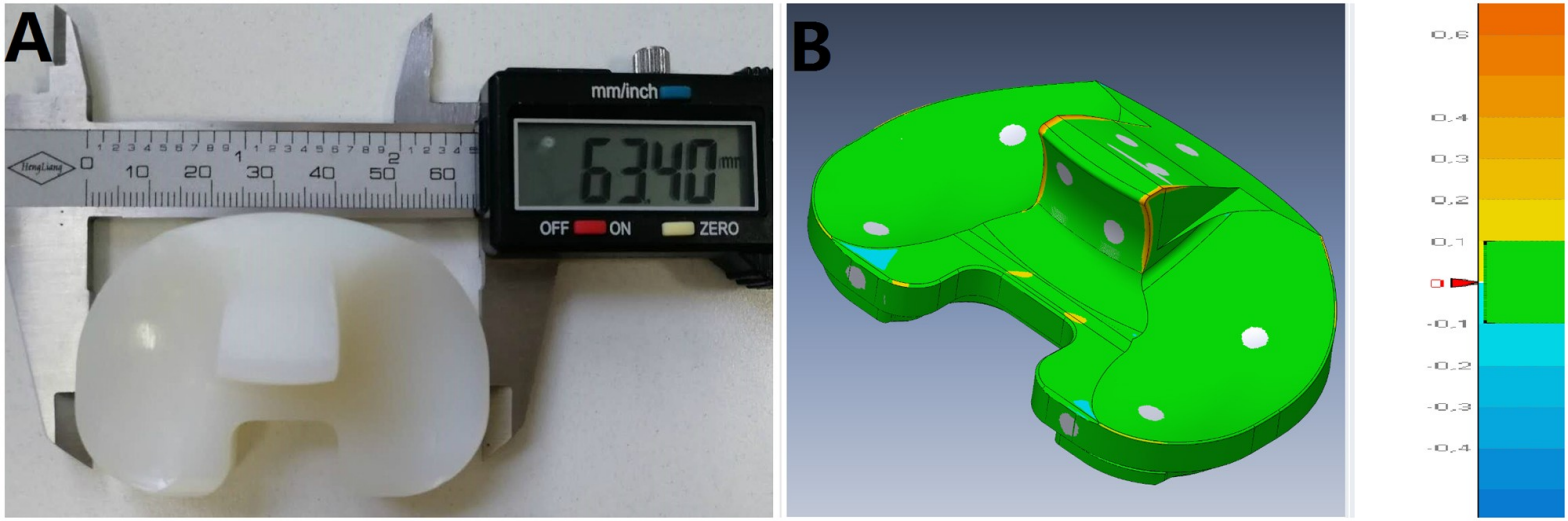
Materials. (A) Retrieved tibial insert from PFC implant; (B) Variations in the articular surface between the 3D model and the retrieved insert were within 0.1mm.

A retrieved knee prosthesis (PFC, Depuy Synthes) of the right knee was used both to construct the finite element model and for experimental work (Fig 4A). An examination of the surfaces of the femoral component and tibia insert found only minor burnishing, abrasion, and scratching, but no obvious cold flow, pitting, embedded metal, delamination or wear through [30]. In the absence of any marked deformities, the minor surface abrasion was deemed acceptable as it would not have any noticeable influence on knee kinematics. A 3D model of the knee joint was constructed in UG software (UG, Siemens NX). Inconsistencies on the articular surface between the 3D model and the physical PFC implant were controlled to be less than 0.1 mm, as shown in Fig 4B.

### FEA models according to ISO14243-3 and ISO14243-1

**Fig 5:**
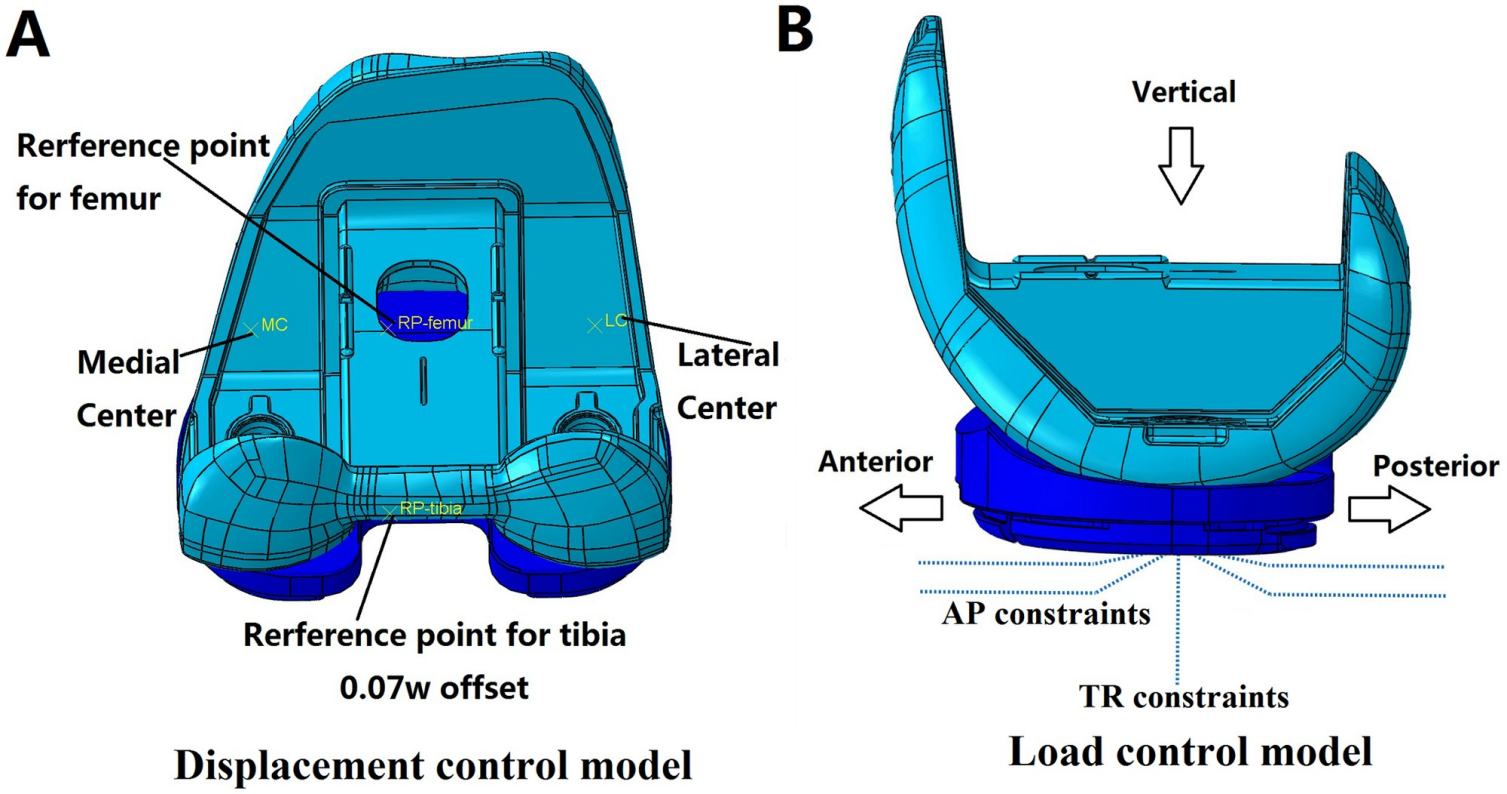
FEA models. (A) Displacement control model according to ISO14243-3; (B) Load control model according to ISO14243-1.

Two finite element models were created according to ISO 14243–3 (displacement control) and ISO 14243–1 (load control) (Fig 5). The two models were identical except the load control model had additional nonlinear connector constraints for limiting AP motion and tibial rotation (TR) according to ISO 14243–1 [12, 14].

The finite element models of the femoral component and tibial insert were developed in Abaqus 2017 (Abaqus, SIMULIA) with the implant geometry being obtained from 3D scans of the retrieved PFC implant. The tibial insert was modeled as deformable elastic polyethylene (Gur1050) with approximately 81,000 C3D10M elements of size 1 mm for the articular surface and 1.6 mm for the remainder of the implant. The femoral component was modeled as a rigid body. A coefficient of friction of 0.04 was adopted based on a prior computational study [31].

The boundary conditions were set according to ISO 14243 standards [11–14]. The loading points on the tibia and femur was offset to the medial side by a distance of 0.07 times the width of the tibial insert (Fig 5A). The mechanical axes described by Grood and Sunday were used [32].

Input curves were applied to the tibia and only a flexion angle was applied to the femoral side. For the displacement controlled models (Fig 5A), the input curves were AP displacement, tibial rotation (TR) angle, flexion angle and compression loading. For the load controlled models (Fig 5B), the input curves were AP load, TR torque, flexion angle and compression loading. Nonlinear constraints in AP and TR directions were imposed on the load controlled model. Along the AP direction, the constraint stiffness was set as 9.3 N/mm if displacement exceeded 2.5 mm, but was 0 N/mm if less than 2.5 mm. For tibial rotation, the constraint stiffness was set as 130±0.01 Nmm/° if angular rotation exceeded ±6°, but was 0 Nmm/° if within 6° [12].

The tibial insert was free to move in the medial-lateral, anterior-posterior, superior-inferior and valgus-varus directions, as well being free to rotate within the tibial component, but was constrained in flexion. In contrast, the femoral component was only permitted to move in flexion. The flexion axis was defined as a line connecting the medial and lateral centers of the posterior femoral radius arc. According to ISO 14243 standards [11–14], the femoral center is defined by considering the condyle of the femoral component to be in contact with an imaginary plane perpendicular to the tibial axis, with the femoral center being the point of intersection of the normal lines of the imaginary plane and running through the contact point under 30° and 60° of flexion.

Five loading conditions were defined based on the directions of AP displacement and TR angle identified in ISO 14243–3, and AP load and TR torque in 14243–1 (Table 1).

**Table 1:** Five loading conditions for FEA models.

Reference standard	Direction of AP	Direction of TR	Control mode
**Modified ISO 14243–3**	positive	positive	Displacement
**ISO 14243–3:2004**	negative	positive	Displacement
**ISO 14243–3:2014**	positive	negative	Displacement
**Modified ISO 14243–1**	positive	positive	Loading
**ISO 14243–1:2009**	negative	positive	Loading

The wear depth was predicted based on Archard’s wear law [33]:

H=KPS (1)

where *H* is wear depth (mm), *K* is wear coefficient (mm3/Nm), *P* is the contact pressure, and *S* is the sliding distance (mm).

The wear coefficient *K* was 2.64*10^−7^ mm^3^/Nm [34–35, 36].

Using the user-defined subroutine UMESHMOTION in Abaqus 2017, the wear depth at each node on the articular surface could be calculated using Archard’s law. According to the calculated wear depth, each node on the surface was moved in the direction normal to the articular surface. An adaptive remeshing procedure was employed to simulate the progression of surface wear. It was calculated for 5 million cycles according to ISO 14243 [11–14]. The volume of wear was calculated by the difference between the initial volume of the tibial insert and its final volume. The tibial insert surface was updated every 500, 000 cycles, which has been shown to only have a difference of between 2.75% to 4.8% with a step size of 125, 000 cycles [35–36]. According to Archard’s wear law, the accuracy of the predicted wear depth depends on the accuracy of the contact pressure and sliding distance. The contact pressure was validated using a Tekscan pressure distribution measuring system (Tekscan Inc, America) (S1 File). The following sections detail the validation of the sliding distance and contact areas using wear contours and feedback curves. The calculation process was developed and validated for use in previous studies on TKA in our laboratory [34–35]. The wear rate was calculated as the average wear volume per million cycles (mm^3^/million cycles).

### Wear contour assessment

**Fig 6:**
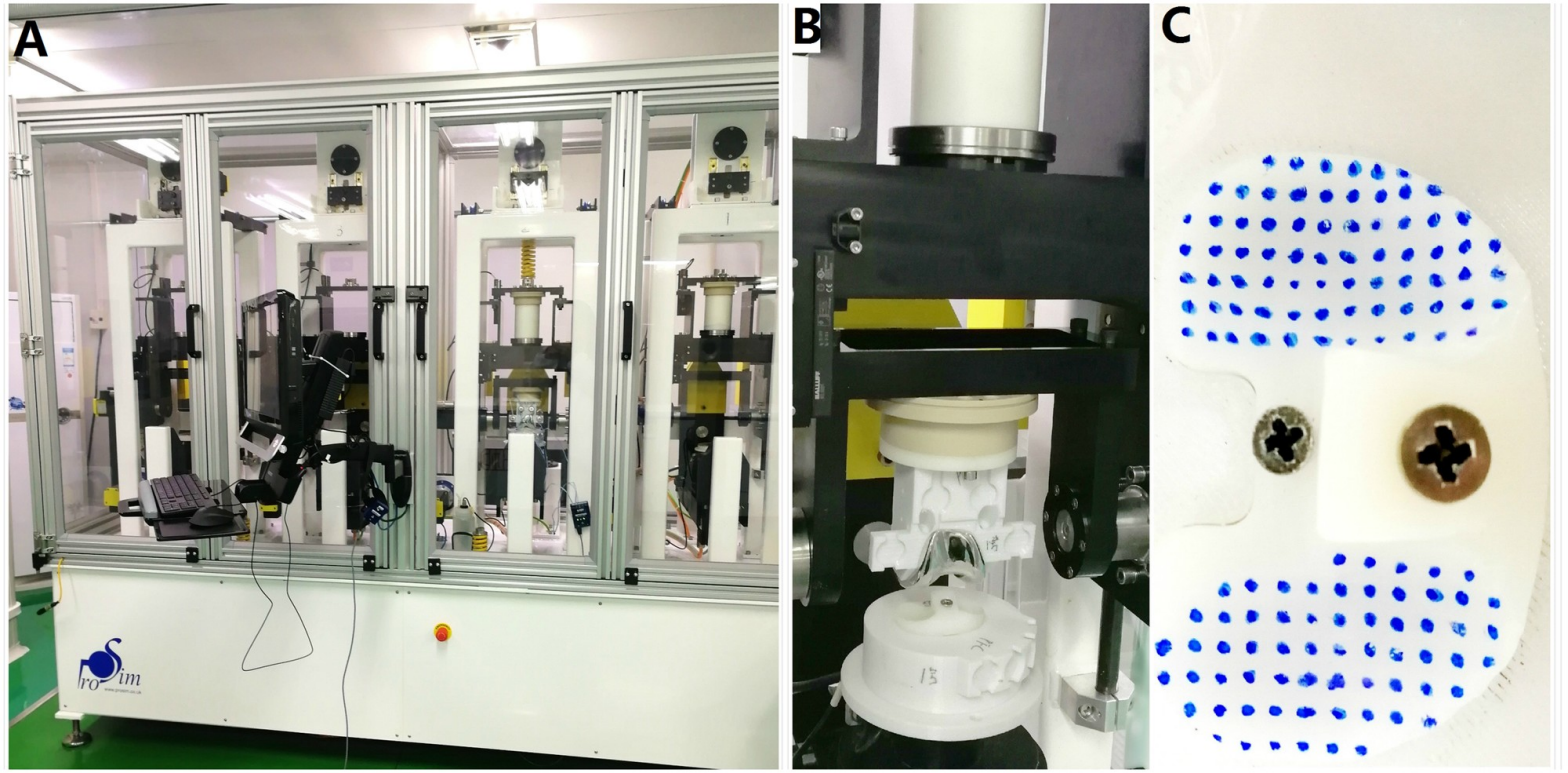
Experimental setup. (A) An electromechanically-controlled Prosim knee simulator; (B) ABS material jigs; (C) Test specimen for assessing wear contours.

As shown in Fig 6A, an electromechanically-controlled knee simulator, Prosim (Prosim, Simulation Solutions), was used to perform the gait movements according to the suggested modifications to ISO 14243–3 with a positive AP displacement (tibia moves anteriorly) and positive TR angle (tibia rotates internally). ABS plastic was used to construct the jigs (Fig 6B). Before the test, the medial and lateral articular surfaces were uniformly coated with small dots using a permanent marker, which was selected as an easy to apply and non-water soluble coating material (Fig 6C). A simple template with uniformly spaced holes was used to ensure consistency with positioning of the holes. 5,000 gait cycles (short-term) were then performed, which had been validated to have similar wear contours with a 5, 000, 000 cycle (full-term) wear test [37].

The loading points on the tibia and femur were offset towards the medial side by a distance of 0.07 times the width of the tibial insert. The flexion axis was the same as the FEA model detailed above. A tolerance of ± 5% of the maximum value and ± 3% of the full cycle time for phasing was required to be maintained for the axial load, flexion angle, AP displacement and TR rotation [14]. The frequency was set at 1 Hz and the test was performed in the air without a medium.

## Results

### FEA model validation

**Fig 7:**
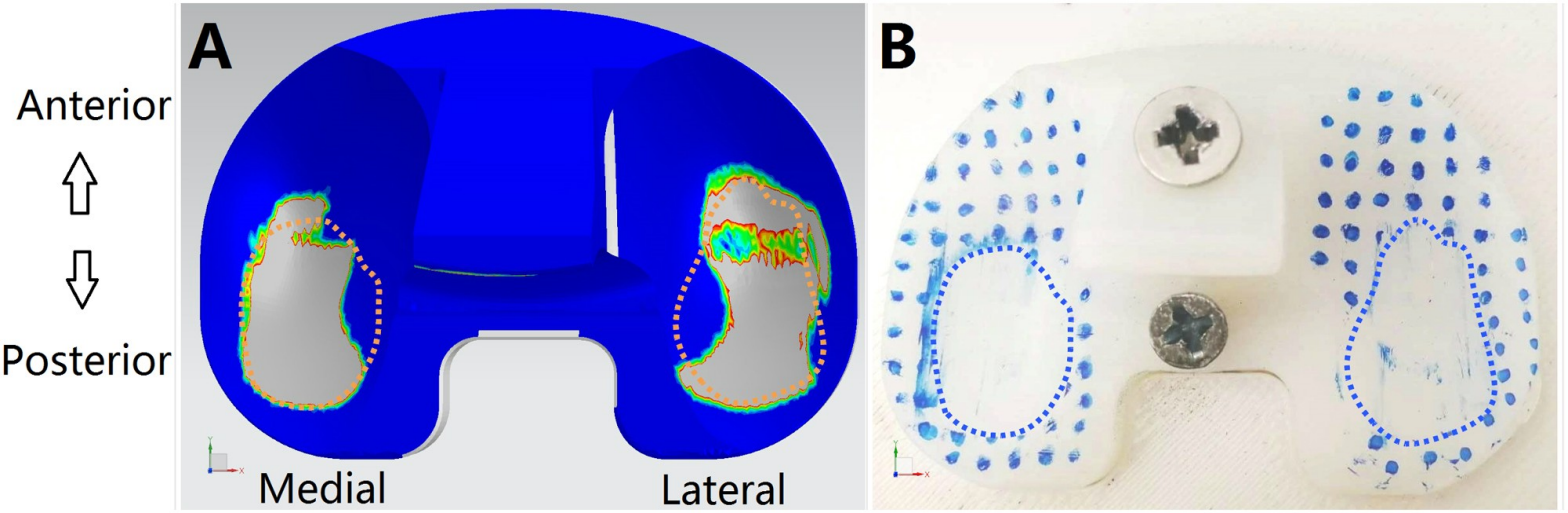
Validation of wear contours. (A) Estimated tibiofemoral wear contours from FEA model; (B) Experimental wear contours from knee simulator.

The wear contours on the tibial insert produced by the FEA model (Fig 7A) were very similar with the wear patterns produced by the knee simulator (Fig 7B).

**Fig 8:**
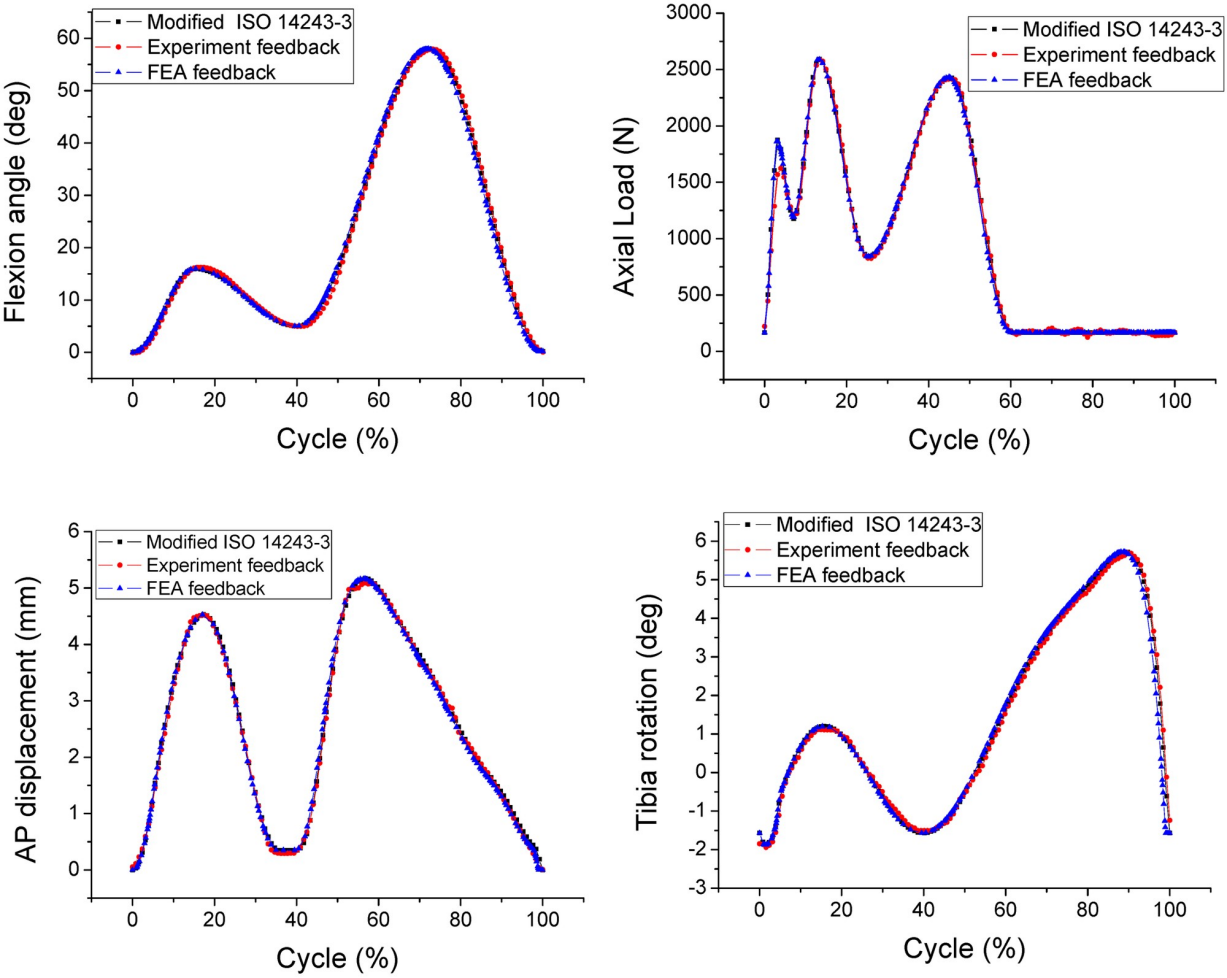
Comparison of gait cycle data among FEA results, experimental results and modified requirements from ISO 14243–3.

The flexion angle, tibial rotation angle, AP displacement, and axial force were recorded from both the FEA model and experimental setup and then compared with the expected inputs from the modified ISO 14243–3 requirements (Fig 8). Fig 8 demonstrates the remarkable similarity between the three sets of data, which confirms the validity of the FEA models introduced in this study.

### Kinematic prediction

**Fig 9:**
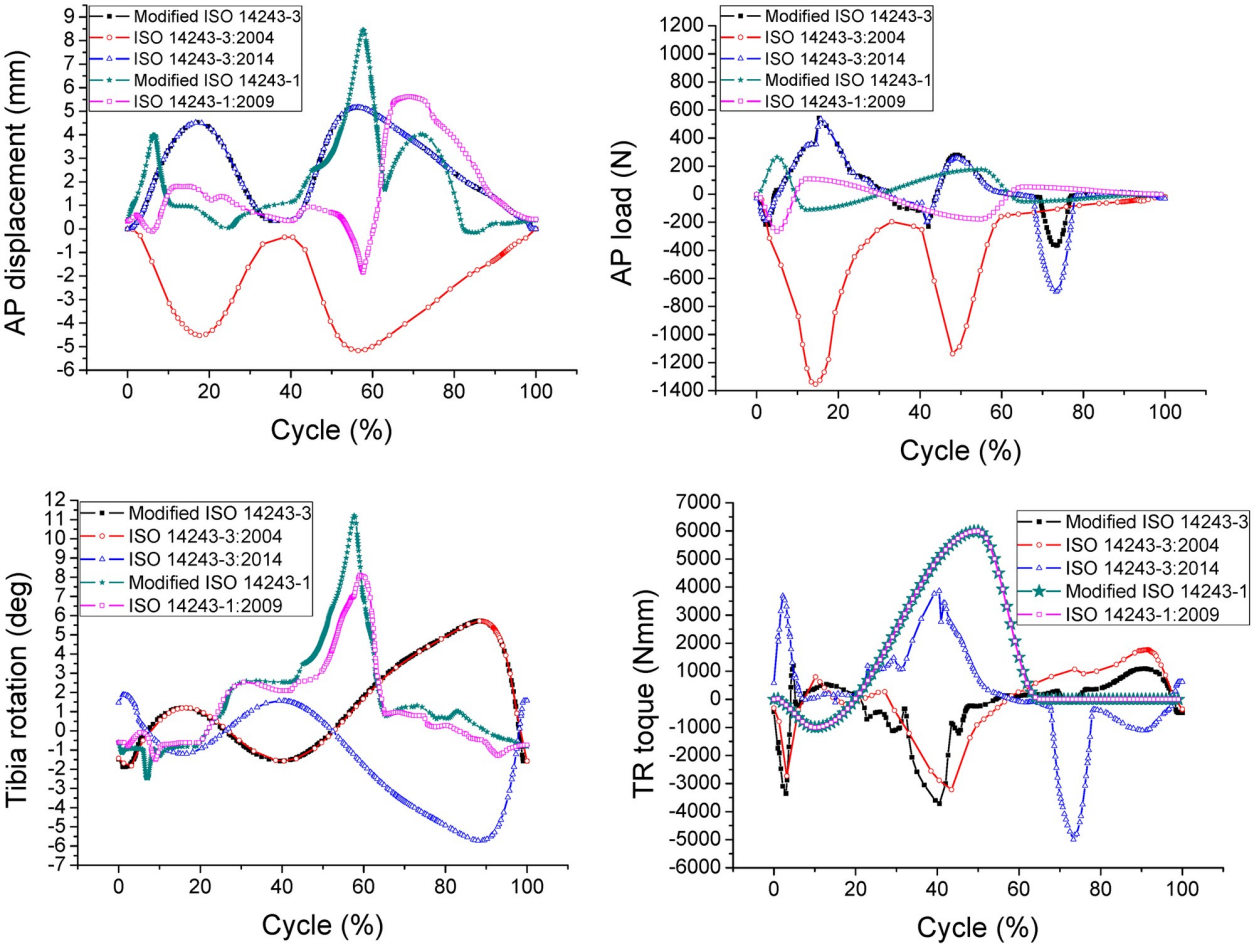
Kinematic results for AP displacement, AP loads, tibial rotation (TR) and TR torque.

For the displacement control models, as shown in Fig 9, the graph of AP displacement plotted according to ISO 14243–3:2004 is an inverse of the graph plotted according to the modified ISO 14243–3. The AP reaction loads are also acting in the opposite direction, but the plot is not symmetrical. The maximum AP loads recorded from the graphs for modified ISO 14243–3, ISO 14243–3:2004 and ISO 14243–3:2014 are 543.6N, -1087.7N and 535.6N, which shows that a negative input for AP displacement produces a greater AP load (-1087.7N). A similar tendency can be seen for tibial rotation whereby the graph of TR angle plotted according to ISO 14243–3:2014 is an inverse of the graph plotted according to the modified ISO 14243–3. The corresponding TR torque also acts in the opposite direction, although is not symmetrical.

For the load control models, as shown in Fig 9, the graph of AP load plotted according to ISO 14243–1:2009 is an inverse of the graph plotted according to the modified ISO 14243–1. A negative AP reaction displacement can be seen in the range of 54%–60% of the gait cycle based on the inverse AP load input (ISO 14243–1:2009).

Between the two ‘modified’ modes of control, the ranges of AP displacement and TR angle are greater for the load control model (modified ISO 14243–1) than the displacement control model (modified ISO 14243–3).

### Tibial insert wear

The wear rate, volumetric wear and maximum wear depth calculated from the FEA models are detailed in Table 2.

**Table 2:** Predicted wear rate, volumetric wear and maximum wear depth.

	Modified ISO 14243–3	ISO14243-3:2004	ISO14243-3:2014	Modified ISO 14243–1	ISO14243-1:2009
**Wear rate (mm**^**3**^**/million)**	8.3	22.64	8.12	12.78	13.64
**Volumetric wear (mm**^**3**^**)**	41.5	113.2	40.6	63.9	68.2
**Maximum wear depth (mm)**	0.598	2.767	0.617	0.547	0.538

For displacement control according to ISO 14243–3, the direction of AP displacement had a marked influence on all three measures of wear, as shown in Table 2; modified ISO 14243–3 to ISO 14243–3:2004. Reversing the direction of AP displacement (modified ISO 14243–3 to ISO 14243–3:2004) increased the wear rate by 272.77% and increased the wear depth by 462.5%. In contrast, changing the direction of the TR angle only had a limited influence on wear; modified ISO 14243–3 to ISO 14243–3:2014.

For load control, reversing the direction of AP load (ISO14243-1:2009) only increased the wear rate by 6.73% over the wear rate calculated according to the modified ISO 14243–1 requirements.

When comparing the two ‘modified’ control modes, the wear rate for load control (modified ISO 14243–1) was 153.98% greater than displacement control (modified ISO 14243–3).

**Fig 10:**
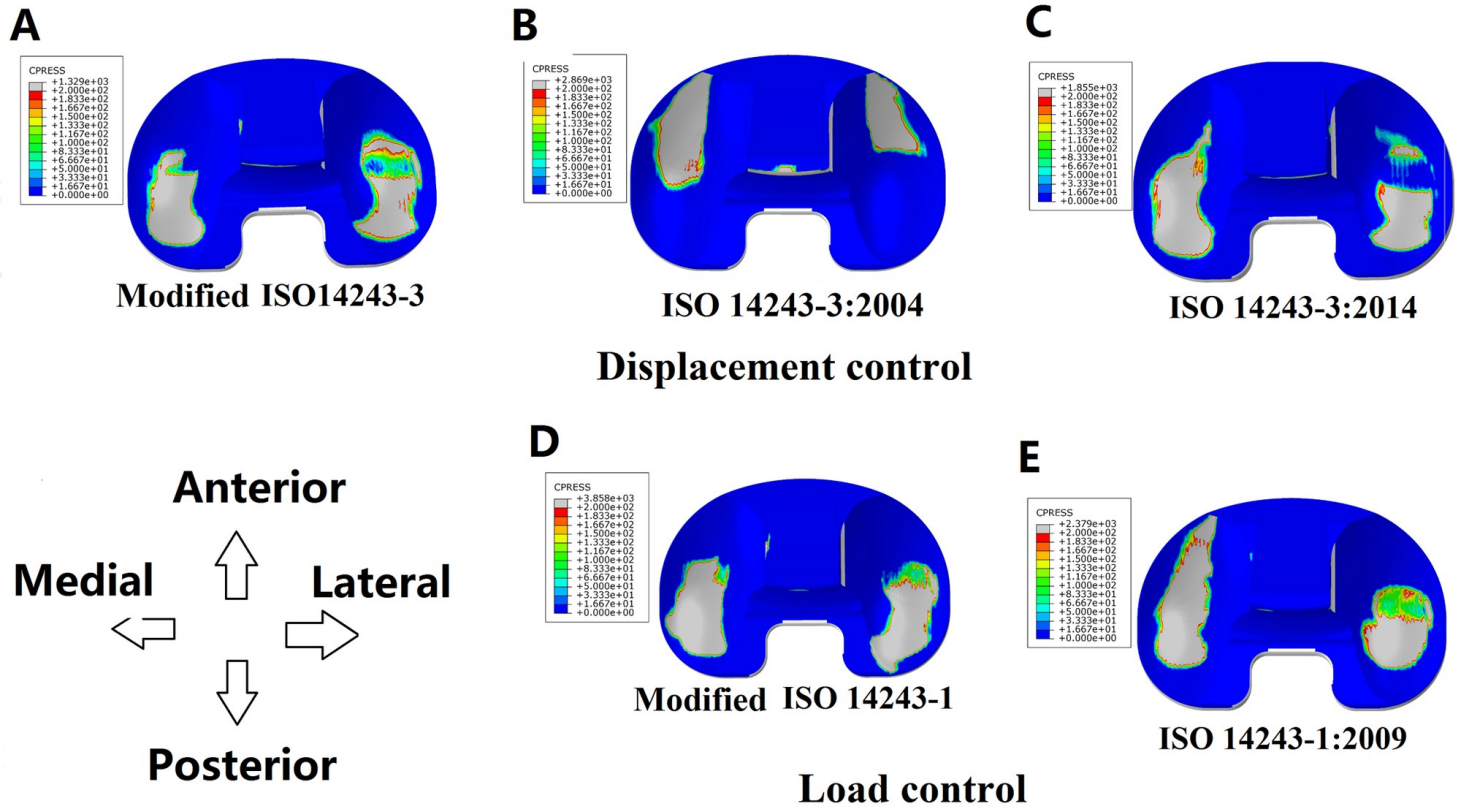
Wear contours from the five FEA models.

Fig 10 shows the wear contours for all models. The wear contours from the modified ISO 14243–3 and modified ISO 14243–1 standards are more consistent in comparison to the other models (both central and slightly posterior on the tibial insert), demonstrating a more natural knee motion [18–23]. In the displacement control models (Fig 10A–10C) it can be seen that the wear contours for the modified ISO 14243–3 model are central and slightly posterior on the tibial insert, whereas wearing on the ISO 14243–3:2004 model occurs in a more anterior position. This is due to the reversed input direction for AP displacement. Between the modified ISO 14243–3 and ISO 14243–3:2014 the direction of AP displacement is the same, but the direction of TR is opposite, which may be the cause of the different wear contours seen in Fig 10A and 10C. For load control (Fig 10D and 10E), the wear contours were positioned more anteriorly or posteriorly depending on the direction of the input for AP load.

## Discussion

The most important finding of this study is that the direction of action of the AP load and TR torque from ISO 14243–1, and AP displacement and TR angle from ISO 14243–3 influences wear on the tibial insert. However, the level of wear varies depending on the design features of the tibial insert and the details of the input curves.

For the displacement control models, reversing the direction of AP displacement (ISO 14243–3:2004) increased the rate of surface wear by 272.77%, which may due to that the shape of the tibial insert with a higher anterior lip. Anterior positioning of the knee contact points results in a greater AP load (Fig 9) and higher wear rates. Reversing the direction of TR angle (ISO 14243–3:2014) does not have such a marked influence on wear, which may due to the symmetrical design of the tibial insert whereby the lateral side is the same as the medial side. This theory may be confirmed by the similar magnitudes of TR torques observed in Fig 9 caused by opposing inputs for TR angle (modified ISO 14243–3 vs. ISO 14243–3:2014). If the insert was not symmetrical, but instead was an anatomical or medial pivot design, reversing the direction of TR angle may have a marked influence on wear.

For the load control models, reversing the AP load also increased the wear rate but to a lesser degree than the displacement controlled models. This may be due to the AP load input curves (Fig 3) having both positive values and negative values. Even when assigned opposing directions for AP loads according to ISO 14243–1:2009 and modified ISO 14243–1, there was less of a difference in the mean AP loads for the load control models, and thus less of a difference in wear (12.78 mm^3^/million and 13.64 mm^3^/million, respectively). In contrast, AP displacement (Fig 2) is either always positive (modified ISO 14243–3) or always negative (ISO 14243–3:2004) during the motion cycles, so changing the direction of AP displacement had a marked influence on wear.

The load control method (modified ISO 14243–1) yielded 153.98% higher wear rates than the displacement control method (modified ISO 14243–3), which may be due to the low conformity design of the posterior articular surface of the tibial insert. Therefore, for the load control models, the AP load and TR torque inputs resulted in a greater range of AP displacement and TR angles than in the displacement control models (Fig 9).

The direction of AP and TR also had a marked influence on the wear contours. In the displacement control models, reversing the direction of AP displacement according to ISO 14243–3 impacted the loading patterns and magnitude, resulting in a marked difference in wear contours. The same phenomenon was observed for the load control models (ISO 14243–1). Overall, the wear contours from the modified ISO 14243–3 and modified ISO 14243–1 were found to be more consistent than the other models. This could be explained by most of the AP displacements and TR angles being in a positive direction (Fig 9).

The FEA model used in this study was fully validated prior to commencing any wear simulations, as highlighted in the following five points. (i) The contact pressure was validated through testing in our laboratory (S1 File). (ii) The wear contours recorded from the FEA models in this study were aligned with experimental findings in this study (Fig 4). (iii) The feedback curves from the FEA models were very similar to the input waveforms. (iv) The finite element-based wear calculations were validated against experimental results published in previous research from our laboratory [34–35]. (v) The wear rates from this study were sensitive to the loading conditions and the changes of wear rates were reasonable based on the above discussion, which further validated the wear models and calculations.

Previous studies on wearing of knee implants [24–25] failed to consider variations in the direction of AP and TR, which has been shown in this study to be a critical factor in determining knee kinematics and the level of wear on the tibial insert. Similarly, Schwenke T et al. [24] reported marked differences in wear rates between displacement control and load control methods.

There are a number of limitations to this study that should be noted. Firstly, the geometrical models and material properties were not sourced directly from the manufacturer, but instead the geometry was based on implant measurements and the material property was defined as UHMWPE Gur 1050. This may lead to slight differences against data published by the original manufacturer. Secondly, for calculating wear depth, Archard’s law does not account for pitting, delamination and third body wear modes, and is limited to predicting abrasive/adhesive wear [38]. Thirdly, neither the effect of creep nor cross-shear was considered in this study. Despite these limitations, it can approximately predict PE wear caused by contact pressure and sliding distance based on Archard’s wear law. Therefore, the current model does provide valuable insight into the influence of AP and TR directions on tibial wear. This study also introduces suggested modifications to the ISO 14243 standards that that closely resemble in vivo situations.

## Conclusions

Altering the directions of AP load and TR torque from those stated in ISO 14243–1, and AP displacement and TR angle in ISO 14243–3 does influence the simulated wear rate and wear contours on the tibial component. However, the extent of this influence varies depending on the design features of the tibial insert and the input parameters. For displacement control, reversing the direction of AP displacement has a marked influence on the simulated wear rate (272.77%), but altering the direction of TR angle has a lesser impact (2.17%). For load control (ISO 14243–1:2009), reversing the AP load only increases the wear rate by 6.73% over the modified ISO 14243–1 parameters put forward in this study. The clinical relevance of this study is that wear tests are affected by the assigned directions of AP and TR. This could influence the estimated longevity of the tibial insert and thus impact the useful life of the implant.

## Supporting information

S1 File Detailed validation of the wear model. (DOCX)

S1 Table AP and TR inputs of ISO 14243–3:2004, ISO 14243–3:2014, modified ISO 14243–3, ISO 14243–1:2009 and modified ISO 14243–1. (DOCX)
