# Supplementary material for: The impact of variations in input directions according to ISO 14243 on wearing of knee prostheses
Source: PLoS One. 2018 Oct 29;13(10):e0206496. doi: 10.1371/journal.pone.0206496 (PMC6205607; doi:10.1371/journal.pone.0206496)
Supplement: S1 File — (DOCX) [file pone.0206496.s001.docx]

**S1 File. Detailed validation of the wear model.**

**1 The accuracy of the predicted wear depth depends on the accuracy of the contact pressure and sliding distance.**

The wear depth was estimated by Archard’s wear law:

$H=KPS$ (1)

where $H$ is wear depth (mm), $K$ is wear coefficient (mm3/Nm), $P$ is the contact pressure, and $S$ is the sliding distance (mm).

So the accuracy of the predicted wear depth mainly depends on the accuracy of the contact pressure and sliding distance. Therefore, the accuracy of contact stress and sliding distance need to be validated firstly.

**2 Accuracy of contact pressure.**

An in vitro experiment was performed on a simplified knee implant using an Instron testing machine (Instron E3000, Instron Inc, America) and Tekscan pressure distribution measuring system (Tekscan, Tekscan Inc, America) under a 1676 N axial load (Fig s1).


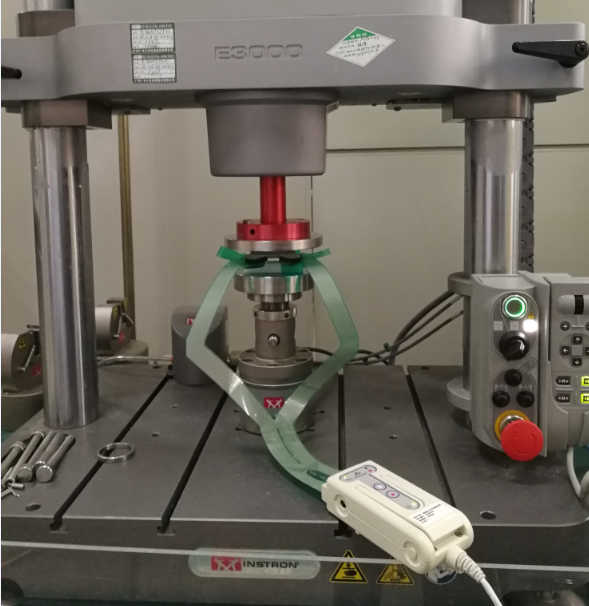


**Fig s1. Measurement of contact stresses using an Instron testing machine and Tekscan.**

| **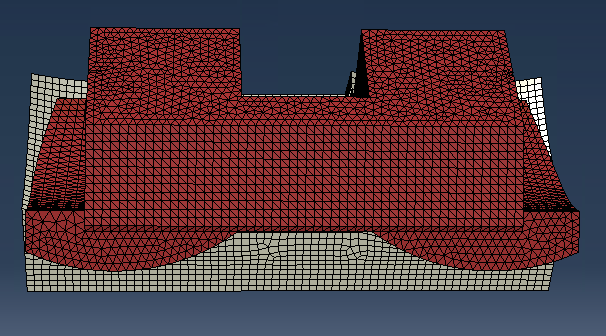**  **Fig s2. Finite element model of simplified knee implant.** | **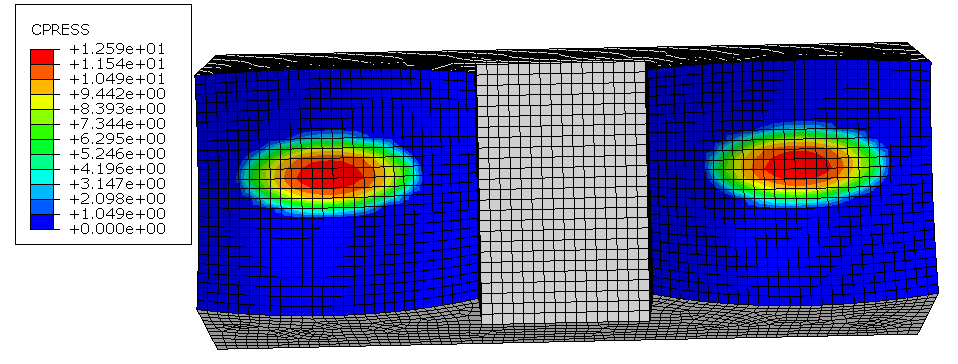**  **Fig s3. Max contact stress from finite element model: 12.59 MPa.** |
| --- | --- |
| **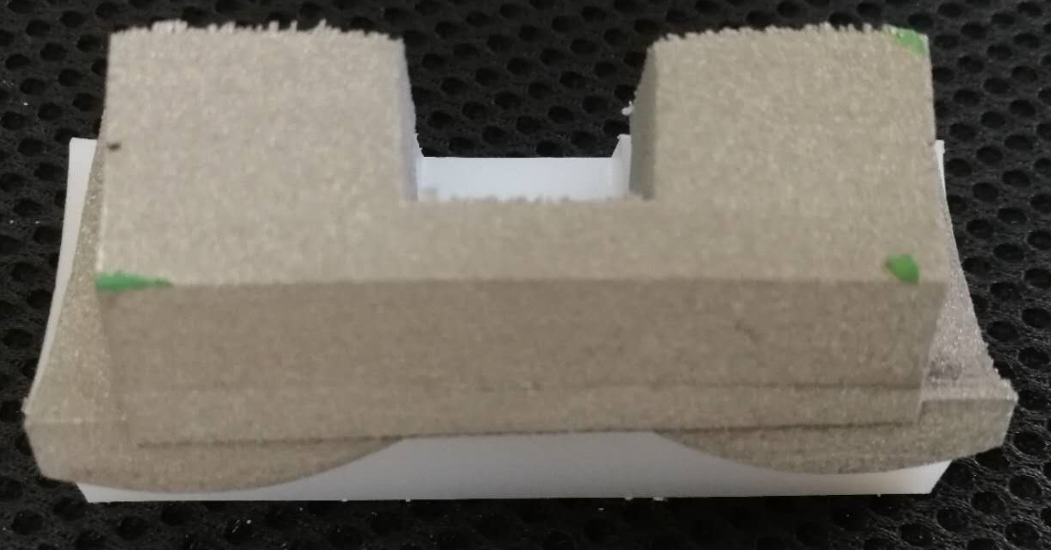**  **Fig s4. Simplified knee implant model to validate the contact property.** | **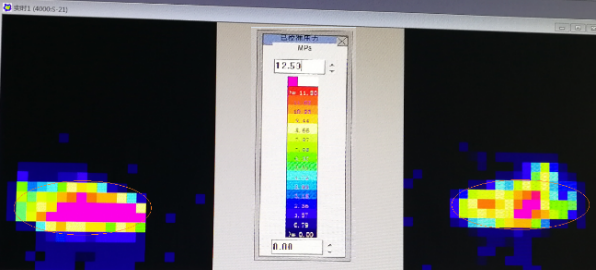**  **Fig s5. Contact stress distribution showing under the a maximum value of 12.59 MPa.** |

The maximum contact pressure of 12.59 MPa was recorded from the finite element model and applied to the experimental model. The resulting contact stress distribution was similar between the experimental model and FEA model (Figs s2-s5).

**3 Accuracy of sliding distance and contact area.**

The wear contours on the tibial insert produced by the FEA model (Fig s6A) were very similar with the wear patterns produced by the knee simulator (Fig s6B).


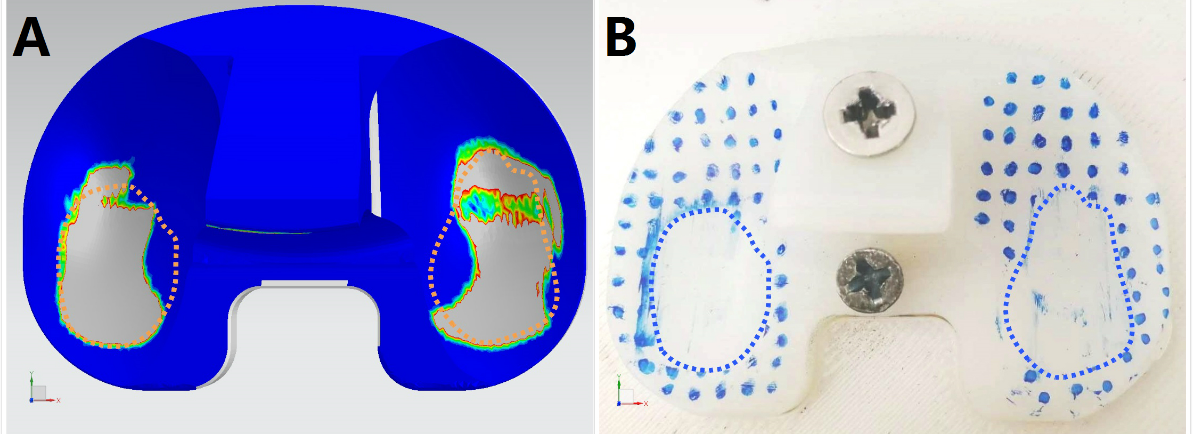


**Fig s6. Validation of wear contours.** (A) Estimated tibiofemoral wear contours from FEA model; (B) Experimental wear contours from knee simulator.

The flexion angle, tibial rotation angle, AP displacement, and axial force were recorded from both the FEA model and experimental setup and then compared with the expected inputs from the modified ISO 14243-3 requirements (Fig s7). Fig s7 demonstrates the remarkable similarity between the three sets of data, which confirms the validity of the FEA models introduced in this study.


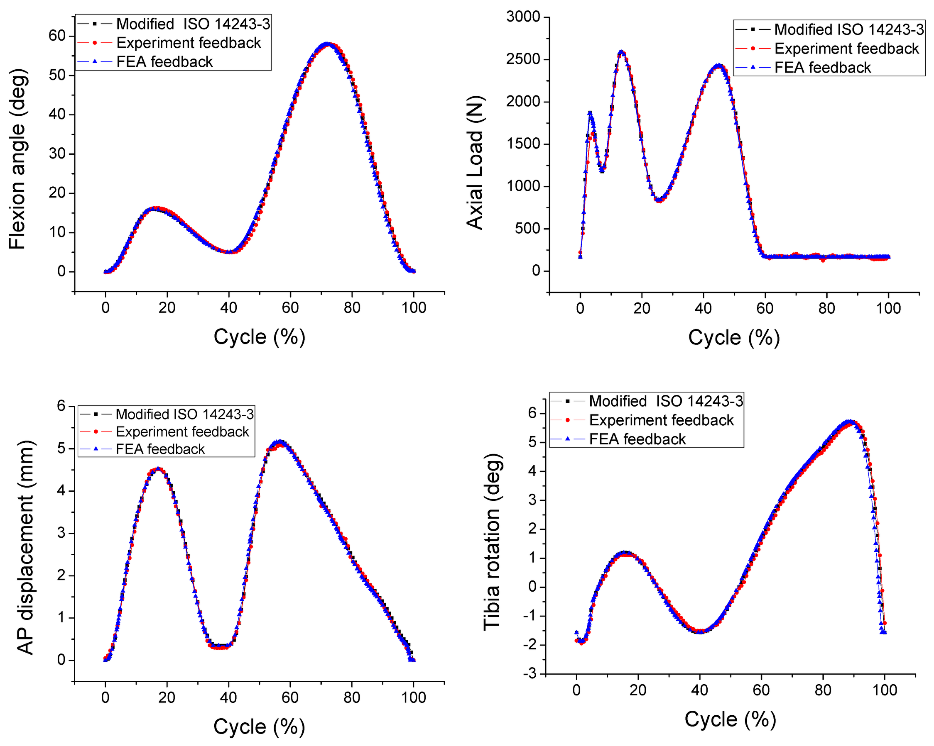


**Fig s7. Comparison of gait cycle data among FEA results, experimental results and modified requirements from ISO 14243-3.**

**4 Wear calculation.**

The calculation process was developed and validated for use in previous studies on TKA in our laboratory using another design of knee implant.

The wear test was performed by Endolab (Endolab, Germany) using another design of knee implant. The finite element model was developed based on the same 3D model and same testing method (ISO 14243-1:2009) (Fig s8).


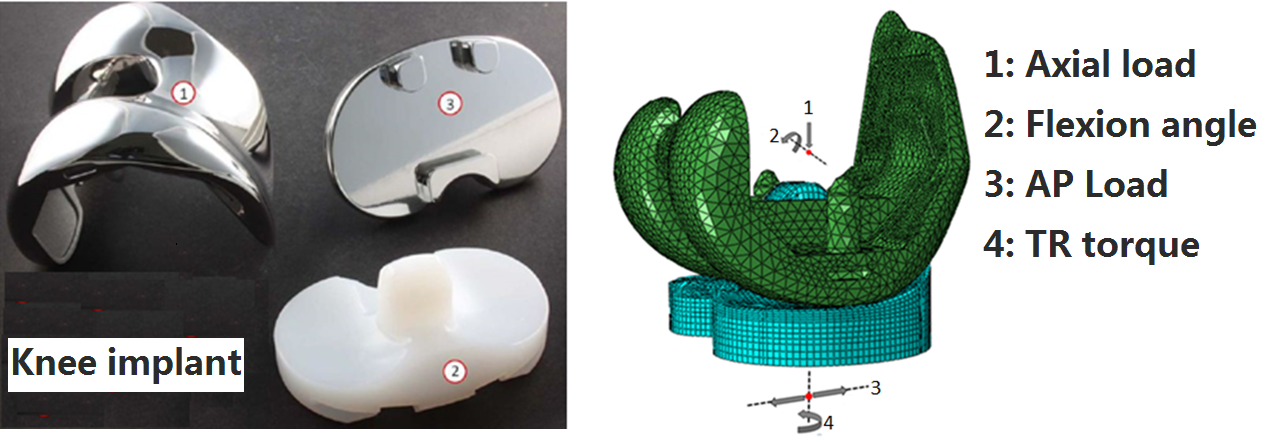


**Fig s8. Finite element model of knee prosthesis using another design of knee implant.**

Mass wear rates, calculated from the in vitro wear test and finite element model, were 14.29±3.19mg/MC (mg/million cycles) and 14.67mg/MC, respectively. These results demonstrate that the wear rates were almost identical between the in vitro model and finite element model (Table s1, Fig s9, and Fig s10).

**Table.s1 The wear data of from experiments.**

| **Samples** | **HF-1** | | **HF-2** | | **HF-3** | | **HF-C** | |
| --- | --- | --- | --- | --- | --- | --- | --- | --- |
| **Cycles** | **mass** | **wear** | **mass** | **wear** | **mass** | **wear** | **mass** | **wear** |
| **(MC)** | **(g)** | **(mg)** | **(g)** | **(mg)** | **(g)** | **(mg)** | **(g)** | **(mg)** |
| **0.0** | 19.90440 | 0.00 | 19.88119 | 0.00 | 19.90512 | 0.00 | 19.85579 | 0.00 |
| **0.5** | 19.90080 | 4.92 | 19.88080 | 1.71 | 19.90334 | 3.10 | 19.85711 | -1.32 |
| **1.0** | 19.89660 | 10.51 | 19.87550 | 8.41 | 19.90066 | 7.17 | 19.85850 | -2.71 |
| **2.0** | 19.88055 | 28.06 | 19.86564 | 19.77 | 19.89001 | 19.32 | 19.86000 | -4.21 |
| **3.0** | 19.85747 | 52.14 | 19.85293 | 33.47 | 19.87381 | 36.52 | 19.86100 | -5.21 |
| **4.0** | 19.84169 | 68.92 | 19.84197 | 45.44 | 19.86357 | 47.77 | 19.86200 | -6.22 |
| **5.0** | 19.82604 | 85.83 | 19.82693 | 61.73 | 19.85407 | 58.52 | 19.86326 | -7.47 |

**Fig s9. Linear wear of inserts from in vitro wear experiment.**


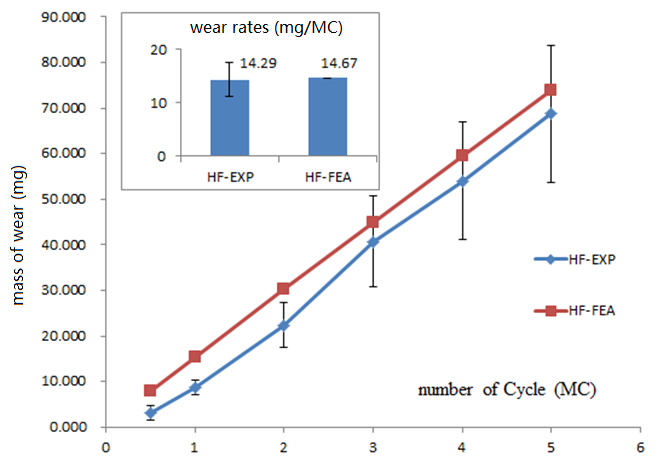


**Fig s10. Linear wear of inserts from in vitro wear experiment and FEA models.**

**5 The wear rates in this study were sensitive to the loading conditions and the changes of wear rates were reasonable based on the information detailed below, which further validated the wear models and calculations.**

**Table s2. Predicted wear rate, volumetric wear and maximum wear depth.**

|  | **Modified ISO 14243-3** | **ISO14243-3:2004** | **ISO14243-3:2014** | **Modified ISO 14243-1** | **ISO14243-1:2009** |
| --- | --- | --- | --- | --- | --- |
| **Wear rate (mm^3^/million)** | 8.3 | 22.64 | 8.12 | 12.78 | 13.64 |
| **Volumetric wear (mm^3^)** | 41.5 | 113.2 | 40.6 | 63.9 | 68.2 |
| **Maximum Wear depth (mm)** | 0.598 | 2.767 | 0.617 | 0.547 | 0.538 |

From Table s2, it can be seen that the wear rates were sensitive to the loading conditions, and the changes of wear rates were reasonable as stated below in the discussion section of the manuscript.

“For the displacement control models, reversing the direction of AP displacement (ISO 14243-3:2004) increased the rate of surface wear by 272.77%, which may due to that the shape of the tibial insert with a higher anterior lip. Anterior positioning of the knee contact points results in a greater AP load (Fig 9) and higher wear rates. Reversing the direction of TR angle (ISO 14243-3:2014) does not have such a marked influence on wear, which may due to the symmetrical design of the tibial insert whereby the lateral side is the same as the medial side. This theory may be confirmed by the similar magnitudes of TR torques observed in Fig. 9 caused by opposing inputs for TR angle (modified ISO 14243-3 vs. ISO 14243-3:2014). If the insert was not symmetrical, but instead was an anatomical or medial pivot design, reversing the direction of TR angle may have a marked influence on wear.

For the load control models, reversing the AP load also increased the wear rate but to a lesser degree than the displacement controlled models. This may be due to the AP load input curves (Fig 3) having both positive values and negative values. Even when assigned opposing directions for AP loads according to ISO 14243-1:2009 and modified ISO 14243-1, there was less of a difference in the mean AP loads for the load control models, and thus less of a difference in wear (12.78 mm3/million and 13.64 mm3/million, respectively). In contrast, AP displacement (Fig 2) is either always positive (modified ISO 14243-3) or always negative (ISO 14243-3:2004) during the motion cycles, so changing the direction of AP displacement had a marked influence on wear.

The load control method (modified ISO 14243-1) yielded 153.98% higher wear rates than the displacement control method (modified ISO 14243-3), which may be due to the low conformity design of the posterior articular surface of the tibial insert. Therefore, for the load control models, the AP load and TR torque inputs resulted in a greater range of AP displacement and TR angles than in the displacement control models (Fig 9).”
