# Supplementary material for: The impact of variations in input directions according to ISO 14243 on wearing of knee prostheses
Source: PLoS One. 2018 Oct 29;13(10):e0206496. doi: 10.1371/journal.pone.0206496 (PMC6205607; doi:10.1371/journal.pone.0206496)
Supplement: S1 Table — (DOCX) [file pone.0206496.s002.docx]

**S1 Table. AP and TR inputs of ISO 14243-3:2004, ISO 14243-3:2014, Modified ISO 14243-3, ISO 14243-1:2009 and Modified ISO 14243-1.**

| **Cycle**  (%) | **ISO 14243-3:2004** | | **ISO 14243-3:2014** | | **Modified ISO 14243-3** | | **ISO 14243-1:2009** | | **Modified ISO 14243-1** | |
| --- | --- | --- | --- | --- | --- | --- | --- | --- | --- | --- |
|  | AP displacement | TR angle | AP displacement | TR angle | AP displacement | TR angle | AP load | TR torque | AP load | TR torque |
|  | mm | ° | mm | ° | mm | ° | N | Nmm | N | Nmm |
| 0 | 0 | -1.57 | 0 | 1.57 | 0 | -1.57 | 0 | 0 | 0 | 0 |
| 1 | -0.04 | -1.87 | 0.04 | 1.87 | 0.04 | -1.87 | -25.31 | 24.5 | 25.31 | 24.5 |
| 2 | -0.21 | -1.87 | 0.21 | 1.87 | 0.21 | -1.87 | -91.56 | -95.5 | 91.56 | -95.5 |
| 3 | -0.49 | -1.7 | 0.49 | 1.7 | 0.49 | -1.7 | -173.44 | -206.1 | 173.44 | -206.1 |
| 4 | -0.84 | -1.36 | 0.84 | 1.36 | 0.84 | -1.36 | -239.69 | -345.5 | 239.69 | -345.5 |
| 5 | -1.32 | -0.54 | 1.32 | 0.54 | 1.32 | -0.54 | -265 | -500 | 265 | -500 |
| 6 | -1.79 | -0.26 | 1.79 | 0.26 | 1.79 | -0.26 | -246.43 | -654.5 | 246.43 | -654.5 |
| 7 | -2.24 | 0.01 | 2.24 | -0.01 | 2.24 | 0.01 | -194.4 | -793.9 | 194.4 | -793.9 |
| 8 | -2.65 | 0.26 | 2.65 | -0.26 | 2.65 | 0.26 | -119.22 | -904.5 | 119.22 | -904.5 |
| 9 | -3.03 | 0.49 | 3.03 | -0.49 | 3.03 | 0.49 | -35.78 | -975.5 | 35.78 | -975.5 |
| 10 | -3.36 | 0.68 | 3.36 | -0.68 | 3.36 | 0.68 | 39.4 | -1000 | -39.4 | -1000 |
| 11 | -3.65 | 0.85 | 3.65 | -0.85 | 3.65 | 0.85 | 91.43 | -989.2 | -91.43 | -989.2 |
| 12 | -3.9 | 0.99 | 3.9 | -0.99 | 3.9 | 0.99 | 110 | -956.9 | -110 | -956.9 |
| 13 | -4.11 | 1.09 | 4.11 | -1.09 | 4.11 | 1.09 | 109.62 | -903.3 | -109.62 | -903.3 |
| 14 | -4.28 | 1.16 | 4.28 | -1.16 | 4.28 | 1.16 | 108.47 | -828.7 | -108.47 | -828.7 |
| 15 | -4.41 | 1.19 | 4.41 | -1.19 | 4.41 | 1.19 | 106.57 | -733.6 | -106.57 | -733.6 |
| 16 | -4.49 | 1.2 | 4.49 | -1.2 | 4.49 | 1.2 | 103.92 | -618.5 | -103.92 | -618.5 |
| 17 | -4.53 | 1.18 | 4.53 | -1.18 | 4.53 | 1.18 | 100.53 | -484.2 | -100.53 | -484.2 |
| 18 | -4.51 | 1.13 | 4.51 | -1.13 | 4.51 | 1.13 | 96.43 | -331.6 | -96.43 | -331.6 |
| 19 | -4.44 | 1.05 | 4.44 | -1.05 | 4.44 | 1.05 | 91.64 | -161.4 | -91.64 | -161.4 |
| 20 | -4.32 | 0.95 | 4.32 | -0.95 | 4.32 | 0.95 | 86.18 | 25.1 | -86.18 | 25.1 |
| 21 | -4.15 | 0.83 | 4.15 | -0.83 | 4.15 | 0.83 | 80.08 | 226.9 | -80.08 | 226.9 |
| 22 | -3.93 | 0.69 | 3.93 | -0.69 | 3.93 | 0.69 | 73.37 | 442.8 | -73.37 | 442.8 |
| 23 | -3.66 | 0.54 | 3.66 | -0.54 | 3.66 | 0.54 | 66.1 | 671.3 | -66.1 | 671.3 |
| 24 | -3.36 | 0.37 | 3.36 | -0.37 | 3.36 | 0.37 | 58.29 | 911 | -58.29 | 911 |
| 25 | -3.02 | 0.2 | 3.02 | -0.2 | 3.02 | 0.2 | 50 | 1160.6 | -50 | 1160.6 |
| 26 | -2.67 | 0.03 | 2.67 | -0.03 | 2.67 | 0.03 | 41.25 | 1418.4 | -41.25 | 1418.4 |
| 27 | -2.31 | -0.15 | 2.31 | 0.15 | 2.31 | -0.15 | 32.11 | 1682.9 | -32.11 | 1682.9 |
| 28 | -1.94 | -0.33 | 1.94 | 0.33 | 1.94 | -0.33 | 22.62 | 1952.5 | -22.62 | 1952.5 |
| 29 | -1.6 | -0.51 | 1.6 | 0.51 | 1.6 | -0.51 | 12.83 | 2225.4 | -12.83 | 2225.4 |
| 30 | -1.28 | -0.67 | 1.28 | 0.67 | 1.28 | -0.67 | 2.8 | 2500 | -2.8 | 2500 |
| 31 | -0.99 | -0.83 | 0.99 | 0.83 | 0.99 | -0.83 | -7.44 | 2774.6 | 7.44 | 2774.6 |
| 32 | -0.74 | -0.98 | 0.74 | 0.98 | 0.74 | -0.98 | -17.81 | 3047.5 | 17.81 | 3047.5 |
| 33 | -0.55 | -1.12 | 0.55 | 1.12 | 0.55 | -1.12 | -28.26 | 3317.1 | 28.26 | 3317.1 |
| 34 | -0.42 | -1.24 | 0.42 | 1.24 | 0.42 | -1.24 | -38.74 | 3581.6 | 38.74 | 3581.6 |
| 35 | -0.35 | -1.34 | 0.35 | 1.34 | 0.35 | -1.34 | -49.19 | 3839.4 | 49.19 | 3839.4 |
| 36 | -0.35 | -1.43 | 0.35 | 1.43 | 0.35 | -1.43 | -59.56 | 4089 | 59.56 | 4089 |
| 37 | -0.35 | -1.49 | 0.35 | 1.49 | 0.35 | -1.49 | -69.8 | 4328.7 | 69.8 | 4328.7 |
| 38 | -0.35 | -1.54 | 0.35 | 1.54 | 0.35 | -1.54 | -79.83 | 4557.2 | 79.83 | 4557.2 |
| 39 | -0.35 | -1.56 | 0.35 | 1.56 | 0.35 | -1.56 | -89.62 | 4773.1 | 89.62 | 4773.1 |
| 40 | -0.35 | -1.57 | 0.35 | 1.57 | 0.35 | -1.57 | -99.11 | 4974.9 | 99.11 | 4974.9 |
| 41 | -0.41 | -1.55 | 0.41 | 1.55 | 0.41 | -1.55 | -108.25 | 5161.4 | 108.25 | 5161.4 |
| 42 | -0.58 | -1.51 | 0.58 | 1.51 | 0.58 | -1.51 | -117 | 5331.6 | 117 | 5331.6 |
| 43 | -0.89 | -1.45 | 0.89 | 1.45 | 0.89 | -1.45 | -125.29 | 5484.2 | 125.29 | 5484.2 |
| 44 | -1.29 | -1.36 | 1.29 | 1.36 | 1.29 | -1.36 | -133.1 | 5618.5 | 133.1 | 5618.5 |
| 45 | -1.81 | -1.26 | 1.81 | 1.26 | 1.81 | -1.26 | -140.37 | 5733.6 | 140.37 | 5733.6 |
| 46 | -2.34 | -1.14 | 2.34 | 1.14 | 2.34 | -1.14 | -147.08 | 5828.7 | 147.08 | 5828.7 |
| 47 | -2.87 | -1 | 2.87 | 1 | 2.87 | -1 | -153.18 | 5903.3 | 153.18 | 5903.3 |
| 48 | -3.37 | -0.84 | 3.37 | 0.84 | 3.37 | -0.84 | -158.64 | 5956.9 | 158.64 | 5956.9 |
| 49 | -3.83 | -0.66 | 3.83 | 0.66 | 3.83 | -0.66 | -163.43 | 5989.2 | 163.43 | 5989.2 |
| 50 | -4.22 | -0.48 | 4.22 | 0.48 | 4.22 | -0.48 | -167.53 | 6000 | 167.53 | 6000 |
| 51 | -4.54 | -0.28 | 4.54 | 0.28 | 4.54 | -0.28 | -170.92 | 5934.4 | 170.92 | 5934.4 |
| 52 | -4.8 | -0.07 | 4.8 | 0.07 | 4.8 | -0.07 | -173.57 | 5740.6 | 173.57 | 5740.6 |
| 53 | -4.97 | 0.15 | 4.97 | -0.15 | 4.97 | 0.15 | -175.47 | 5427.1 | 175.47 | 5427.1 |
| 54 | -5.08 | 0.38 | 5.08 | -0.38 | 5.08 | 0.38 | -176.62 | 5007.4 | 176.62 | 5007.4 |
| 55 | -5.14 | 0.61 | 5.14 | -0.61 | 5.14 | 0.61 | -177 | 4500 | 177 | 4500 |
| 56 | -5.17 | 0.85 | 5.17 | -0.85 | 5.17 | 0.85 | -171.4 | 3927.1 | 171.4 | 3927.1 |
| 57 | -5.16 | 1.08 | 5.16 | -1.08 | 5.16 | 1.08 | -155.13 | 3313.6 | 155.13 | 3313.6 |
| 58 | -5.12 | 1.32 | 5.12 | -1.32 | 5.12 | 1.32 | -129.8 | 2686.4 | 129.8 | 2686.4 |
| 59 | -5.05 | 1.55 | 5.05 | -1.55 | 5.05 | 1.55 | -97.88 | 2072.9 | 97.88 | 2072.9 |
| 60 | -4.96 | 1.78 | 4.96 | -1.78 | 4.96 | 1.78 | -62.5 | 1500 | 62.5 | 1500 |
| 61 | -4.86 | 2 | 4.86 | -2 | 4.86 | 2 | -27.12 | 992.6 | 27.12 | 992.6 |
| 62 | -4.74 | 2.22 | 4.74 | -2.22 | 4.74 | 2.22 | 4.8 | 572.9 | -4.8 | 572.9 |
| 63 | -4.62 | 2.43 | 4.62 | -2.43 | 4.62 | 2.43 | 30.13 | 259.4 | -30.13 | 259.4 |
| 64 | -4.49 | 2.64 | 4.49 | -2.64 | 4.49 | 2.64 | 46.4 | 65.6 | -46.4 | 65.6 |
| 65 | -4.36 | 2.83 | 4.36 | -2.83 | 4.36 | 2.83 | 52 | 0 | -52 | 0 |
| 66 | -4.24 | 3.02 | 4.24 | -3.02 | 4.24 | 3.02 | 51.9 | 0 | -51.9 | 0 |
| 67 | -4.11 | 3.19 | 4.11 | -3.19 | 4.11 | 3.19 | 51.58 | 0 | -51.58 | 0 |
| 68 | -3.99 | 3.36 | 3.99 | -3.36 | 3.99 | 3.36 | 51.06 | 0 | -51.06 | 0 |
| 69 | -3.87 | 3.52 | 3.87 | -3.52 | 3.87 | 3.52 | 50.34 | 0 | -50.34 | 0 |
| 70 | -3.75 | 3.67 | 3.75 | -3.67 | 3.75 | 3.67 | 49.43 | 0 | -49.43 | 0 |
| 71 | -3.63 | 3.81 | 3.63 | -3.81 | 3.63 | 3.81 | 48.32 | 0 | -48.32 | 0 |
| 72 | -3.51 | 3.94 | 3.51 | -3.94 | 3.51 | 3.94 | 47.03 | 0 | -47.03 | 0 |
| 73 | -3.38 | 4.07 | 3.38 | -4.07 | 3.38 | 4.07 | 45.58 | 0 | -45.58 | 0 |
| 74 | -3.25 | 4.2 | 3.25 | -4.2 | 3.25 | 4.2 | 43.97 | 0 | -43.97 | 0 |
| 75 | -3.11 | 4.32 | 3.11 | -4.32 | 3.11 | 4.32 | 42.21 | 0 | -42.21 | 0 |
| 76 | -2.96 | 4.44 | 2.96 | -4.44 | 2.96 | 4.44 | 40.32 | 0 | -40.32 | 0 |
| 77 | -2.82 | 4.56 | 2.82 | -4.56 | 2.82 | 4.56 | 38.32 | 0 | -38.32 | 0 |
| 78 | -2.67 | 4.68 | 2.67 | -4.68 | 2.67 | 4.68 | 36.22 | 0 | -36.22 | 0 |
| 79 | -2.53 | 4.8 | 2.53 | -4.8 | 2.53 | 4.8 | 34.03 | 0 | -34.03 | 0 |
| 80 | -2.38 | 4.92 | 2.38 | -4.92 | 2.38 | 4.92 | 31.79 | 0 | -31.79 | 0 |
| 81 | -2.25 | 5.04 | 2.25 | -5.04 | 2.25 | 5.04 | 29.49 | 0 | -29.49 | 0 |
| 82 | -2.12 | 5.16 | 2.12 | -5.16 | 2.12 | 5.16 | 27.17 | 0 | -27.17 | 0 |
| 83 | -2 | 5.29 | 2 | -5.29 | 2 | 5.29 | 24.83 | 0 | -24.83 | 0 |
| 84 | -1.89 | 5.4 | 1.89 | -5.4 | 1.89 | 5.4 | 22.51 | 0 | -22.51 | 0 |
| 85 | -1.79 | 5.51 | 1.79 | -5.51 | 1.79 | 5.51 | 20.21 | 0 | -20.21 | 0 |
| 86 | -1.7 | 5.6 | 1.7 | -5.6 | 1.7 | 5.6 | 17.97 | 0 | -17.97 | 0 |
| 87 | -1.61 | 5.68 | 1.61 | -5.68 | 1.61 | 5.68 | 15.78 | 0 | -15.78 | 0 |
| 88 | -1.51 | 5.72 | 1.51 | -5.72 | 1.51 | 5.72 | 13.68 | 0 | -13.68 | 0 |
| 89 | -1.41 | 5.72 | 1.41 | -5.72 | 1.41 | 5.72 | 11.68 | 0 | -11.68 | 0 |
| 90 | -1.3 | 5.66 | 1.3 | -5.66 | 1.3 | 5.66 | 9.79 | 0 | -9.79 | 0 |
| 91 | -1.17 | 5.53 | 1.17 | -5.53 | 1.17 | 5.53 | 8.03 | 0 | -8.03 | 0 |
| 92 | -1.03 | 5.3 | 1.03 | -5.3 | 1.03 | 5.3 | 6.42 | 0 | -6.42 | 0 |
| 93 | -0.89 | 4.94 | 0.89 | -4.94 | 0.89 | 4.94 | 4.97 | 0 | -4.97 | 0 |
| 94 | -0.74 | 4.44 | 0.74 | -4.44 | 0.74 | 4.44 | 3.68 | 0 | -3.68 | 0 |
| 95 | -0.62 | 3.75 | 0.62 | -3.75 | 0.62 | 3.75 | 2.57 | 0 | -2.57 | 0 |
| 96 | -0.51 | 2.83 | 0.51 | -2.83 | 0.51 | 2.83 | 1.66 | 0 | -1.66 | 0 |
| 97 | -0.43 | 1.64 | 0.43 | -1.64 | 0.43 | 1.64 | 0.94 | 0 | -0.94 | 0 |
| 98 | -0.34 | 0.13 | 0.34 | -0.13 | 0.34 | 0.13 | 0.42 | 0 | -0.42 | 0 |
| 99 | 0 | -1.57 | 0 | 1.57 | 0 | -1.57 | 0.1 | 0 | -0.1 | 0 |
